# Chronic Pancreatitis and Pancreas Divisum: A Case Report of Recurrent Management Challenges

**DOI:** 10.7759/cureus.82313

**Published:** 2025-04-15

**Authors:** Shivangini Duggal, Ozioma Akahara, Claudia Didia

**Affiliations:** 1 Internal Medicine, Texas Tech University of Health Sciences, El Paso, USA; 2 Internal Medicine, Texas Tech University Health Sciences Center El Paso Paul L. Foster School of Medicine, El Paso, USA

**Keywords:** acute-on-chronic pancreatitis, endoscopy ercp, pancreatic divisum, recurrent acute pancreatitis, recurrent pancreatitis

## Abstract

Pancreas divisum, resulting from incomplete fusion of the pancreatic ducts during development, disrupts normal drainage and can lead to recurrent acute and chronic pancreatitis. This report presents a case of a 46-year-old male with chronic necrotizing pancreatitis secondary to pancreas divisum. The patient experienced multiple hospital admissions and underwent a cholecystectomy before the underlying etiology, pancreas divisum, was identified after six hospitalizations. This case highlights the diagnostic challenges of recurrent pancreatitis, emphasizing the importance of considering congenital pancreatic anomalies in patients with unexplained or refractory disease. It also underscores the need for a systematic approach to evaluating recurrent pancreatitis to avoid delays in diagnosis and unnecessary interventions. Pancreas divisum is associated with recurrent pancreatitis in a subset of patients. While endoscopic retrograde cholangiopancreatography remains the gold standard for diagnosis and intervention, non-invasive imaging such as magnetic resonance cholangiopancreatography is preferred for initial diagnosis. Endoscopic treatment, including minor papilla papillotomy and stenting, is typically effective for symptomatic cases. However, surgery may be necessary when these methods fail.

## Introduction

Pancreatitis is a common gastrointestinal cause of hospitalization, stemming from inflammation of the pancreas. The most common causes of acute pancreatitis are gallstones, alcohol use, hypercalcemia, hyperparathyroidism, drug-induced causes, smoking, hypertriglyceridemia, and autoimmune conditions. Genetic factors, including mutations in the cystic fibrosis transmembrane conductance regulator gene (CFTR) and cationic trypsinogen gene (PRSS1), are rare causes of pancreatitis. It may also be associated with structural causes, including pancreas divisum and sphincter of Oddi dysfunction [1,2]. It arises secondary to the abnormal damaging activity of proteolytic enzymes within the pancreatic acinar cells [3]. Diagnosis of acute pancreatitis follows the Atlanta classification, which requires at least two of the following to be present: classical abdominal pain (localized to the epigastrium and often radiating to the back), serum lipase or amylase at least three times the upper limit of normal, or specific findings of acute pancreatitis on computed tomography (CT) or magnetic resonance imaging (MRI) [4,5].

Pancreas divisum is the result of a failure in fusion and rotation of the ventral and dorsal pancreatic buds during the sixth and seventh weeks of gestation, leading to either total emptying via the accessory pancreatic duct or a restricted connection between the ventral and dorsal pancreatic ducts [6]. Our patient represents a unique case in which the diagnosis of pancreas divisum was made after eight episodes of pancreatitis. He underwent cholecystectomy, multiple ERCPs (endoscopic retrograde cholangiopancreatography), and imaging before pancreas divisum was identified on MRI.

## Case presentation

**Figure 1:**
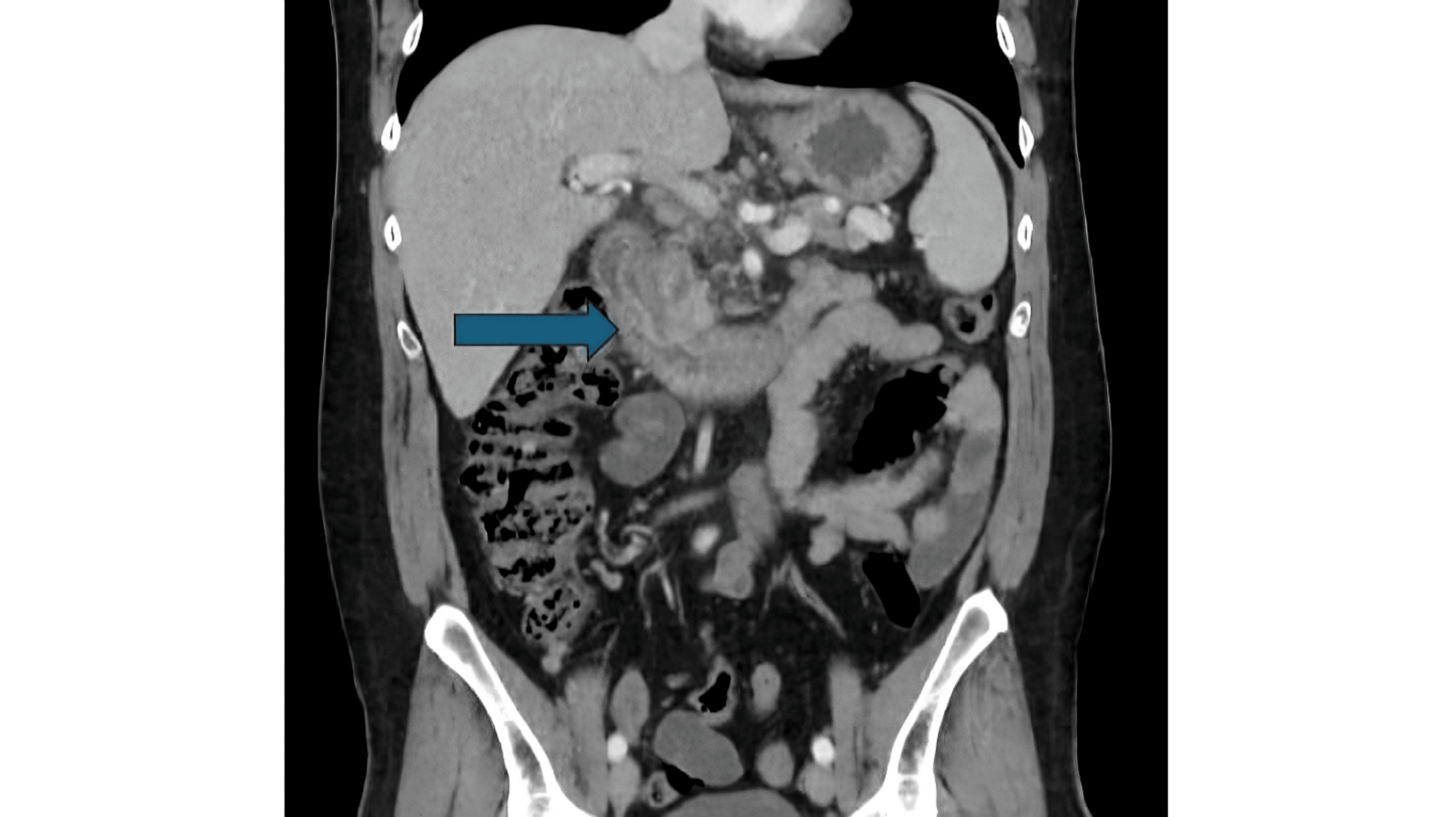
Computed tomography of the abdomen showing pancreatic ductal dilation prompting an MRCP. MRCP: magnetic resonance cholangiopancreatography.

**Figure 2:**
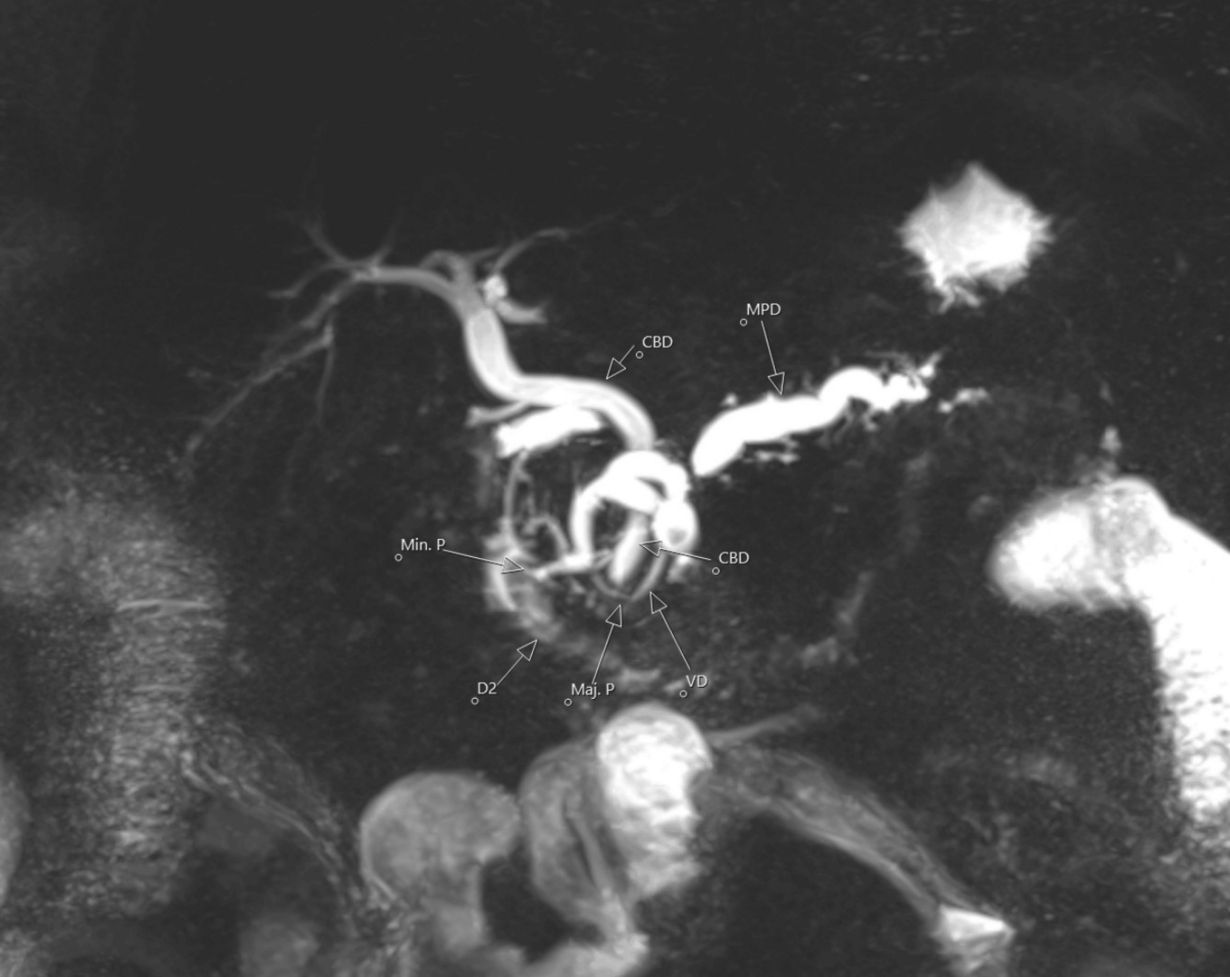
MRCP demonstrating pancreas divisum MRCP (magnetic resonance cholangiopancreatography) coronal MIP (maximum intensity projection) image in a patient with a history of recurrent pancreatitis. There is significant alteration of the baseline variant anatomy due to prior necrotizing pancreatitis, resulting in discontinuity of the main pancreatic duct (MPD) and dilated side branches at the neck of the pancreas. The MPD is noted to insert at the minor papilla, separate from the common bile duct insertion into the major papilla, consistent with pancreatic divisum.

A 46-year-old male with a known history of chronic necrotizing pancreatitis status post cholecystectomy with unknown etiology presented to the emergency department (ED) with abdominal pain for a duration of one day. On presentation, he endorsed diffuse abdominal pain following a meal and several episodes of emesis, nausea, shortness of breath, and cough. Earliest vital signs were consistent with an afebrile oral temperature of 36.6 °C, normal heart rate of 83, tachypnea at 22 breaths per minute, hypertensive blood pressure at 164/91 mmHg, and oxygen saturation of 100% on room air. Initial physical examination was notable for tenderness in the epigastrium, right upper quadrant, and left upper quadrant to palpation. Initial laboratory studies showed neutrophilic leukocytosis, elevated lipase at 5,952 IU/L, mild hypokalemia, acidosis, and minimal hyperglycemia (Table 1). Abdominal CT (Figure 1) was obtained and revealed acute-on-chronic pancreatitis and ductal dilation suggestive of pancreas divisum. Pancreas divisum was confirmed by magnetic resonance cholangiopancreatography (MRCP) (Figure 2) and was identified as the likely cause of the patient’s recurrent chronic pancreatitis. Inflammatory markers, erythrocyte sedimentation rate (ESR) and C-reactive protein (CRP), were not obtained as the primary diagnosis had already been established. The patient’s symptoms of nausea were managed with ondansetron 4 mg, and following administration of morphine 2 mg, acetaminophen 650 mg, and hydrocodone-acetaminophen 5 mg/325 mg as needed for pain, the patient became normotensive with blood pressure at 122/71 mmHg. With adequate resolution of pain and nausea, the patient denied any persistent cough or shortness of breath. On discharge, the patient’s laboratory values were overall unremarkable, with hyperphosphatemia at 5.2 mg/dL.

**Table 1:** Initial laboratory work-up WBC: white blood cell count, RBC: red blood cell count, HGB: hemoglobin, HCT: hematocrit, MCV: mean corpuscular volume, BUN: blood urea nitrogen, GOT (AST): glutamic oxaloacetic transaminase (aspartate aminotransferase), ALK PHOS: alkaline phosphatase, GPT (ALT): glutamic pyruvic transaminase (alanine aminotransferase).

Serum Test	Result	Normal Range
WBC	13.54 × 10^3^/UL High	(4.50–11.00) × 10^3^/UL
RBC	5.2 × 10^6^/UL	(4.20–5.90) × 10^6^/UL
HGB	15.3 G/DL	12.0–16.0 G/DL
HCT	43.2%	38.0–47.0%
MCV	83.1 fL	82.0–98.0 fL
PLT	158 × 10^3^/UL	(150–450) × 10^3^/UL
Neutrophil absolute count	10.29 × 10^3^/UL (High)	(2.00–7.80) × 10^3^/UL
Lymphocyte absolute count	2.34 × 10^3^/UL	(1.00–4.80) × 10^3^/UL
Sodium serum	138 mmol/L	135–145 mmol/L
Potassium serum	3.4 mmol/L (Low)	3.5–5.1 mmol/L
Chloride serum	113 mmol/L (High)	98–107 mmol/L
HCO_3_	18 mmol/L (Low)	22–30 mmol/L
Anion gap	7 mmol/L	5–19 mmol/L
Glucose serum	109 mg/dL (High)	74–106 mg/dL
Bun serum	14 mg/dL	9–20 mg/dL
Creatinine	0.6 mg/dL (Low)	0.66–1.25 mg/dL
Calcium serum	9.3 mg/dL	8.4–10.2 mg/dL
Albumin serum	4.3 g/dL	3.5–5.0 g/dL
Protein serum	7.4 g/dL	6.3–8.2 g/dL
Total bilirubin	1 mg/dL	0.2–1.3 mg/dL
GOT (AST)	21 IU/L	17–59 IU/L
Alkaline phosphatase	98 IU/L	38–126 IU/L
GPT (ALT)	14 IU/L	0–50 IU/L
Lipase result	5952 IU/L (High)	23–300 IU/L
Troponin I	<0.012 ng/mL	0.000–0.034 ng/mL
Glycohemoglobin A1C	5 %	<5.7%

Although the patient had undergone multiple prior abdominal CT scans during previous episodes of pancreatitis, the presence of a pancreas divisum had not been identified until this admission. This is likely attributable to the severely inflamed pancreatic environment during those episodes, which may have limited the sensitivity of cross-sectional imaging in detecting subtle ductal anomalies such as pancreas divisum. During this admission, an abdominal CT scan was again performed, as it remains the clinical standard for imaging in cases of suspected acute pancreatitis.

Of note, the patient had seven prior admissions to the hospital for similar complaints, and the pancreas divisum was discovered on the eighth admission. During the first admission, the patient endorsed a three-day history of sharp abdominal pain, rated 10 out of 10 in severity and radiating toward the back. He reported nausea and vomiting but denied any history of gallbladder-related pathologies. He had no past medical or surgical history at the time. Physical examination was consistent with mild-to-moderate tenderness to palpation in the epigastric and periumbilical regions. Abdominal CT revealed findings of prominent acute pancreatitis, with peripancreatic hypodense edema and marked peripancreatic and adjacent stranding. Air- and fluid-filled mildly dilated small bowel loops were also seen, excluding the distal ileum at the level of the right pelvis, concerning for partial small bowel obstruction versus ileus associated with pancreatitis. He underwent a cholecystectomy, as that was the presumed cause of his pancreatitis. However, he was readmitted due to persistent unresolved epigastric pain. CT abdomen and pelvis (CTAP) showed necrotizing pancreatitis with a walled-off necrotic collection measuring 14 × 7 × 7 cm. Upper endoscopic ultrasound (EUS) showed diffuse erythematous gastric mucosa and a pancreatic pseudocyst, leading to cystogastrostomy with AXIOS stent placement.

During his third admission for similar complaints, laboratory findings included elevated lipase, ESR, and CRP at 3,136 IU/L, 17 mm/hr, and 6.4 mg/dL, respectively. CTAP showed improvement in necrotizing pancreatitis with a walled-off collection and migration of the previously placed AXIOS stent into the gastric lumen. Upper endoscopy revealed partial sealing of the cystogastrostomy tract, and the AXIOS stent was removed. Patency of the tract was restored, and double pigtail stents were placed.

The patient’s frequency of admissions for pancreatitis decreased subsequently. Prior to this intervention, he had been admitted five times in five months; after the procedure, he did not return to the emergency department for similar concerns until seven months later. Upon discharge, he was started on pancrelipase, followed by a low-fat diet, and he abstained from alcohol and smoking. He is currently being followed as an outpatient in the gastroenterology clinic and is scheduled for an endoscopic pancreatic sphincterotomy.

## Discussion

Pancreas divisum is classified into three types: type 1 (the most common), in which the ventral and dorsal pancreatic ducts are completely divided; type 2, in which the ventral pancreatic duct is absent; and type 3, in which a thin duct between the two systems is present [7]. Pancreas divisum represents the main pancreatic anatomical variation, with an incidence of 4.5%, as reported in a recent review. Approximately 5% of patients with pancreas divisum develop symptomatic disease. These patients may present with recurrent acute pancreatitis, chronic pancreatitis, or chronic abdominal pain. The overall prevalence of pancreas divisum is 18%, and in patients who present with pancreatitis, the prevalence is 30% [8]. There has been a reported increase in the incidence and hospitalization rates of acute pancreatitis, with global rates rising by 3.07% per year [9]. The incidence rate of chronic pancreatitis ranges between 5 to 12 per 100,000 individuals per year, with a prevalence of 50 per 100,000 [10]. This patient experienced multiple episodes of pancreatitis over several years, leading to repeated hospitalizations and invasive interventions, including cholecystectomy, multiple ERCPs, and endoscopic stent placements, before the underlying anatomical cause was identified via MRCP. Despite the availability of advanced imaging techniques, the diagnosis of pancreas divisum was delayed, resulting in substantial morbidity.

The suggested mechanism for pancreatitis in pancreas divisum cases is linked to the anatomical abnormality. In these instances, pancreatic drainage predominantly occurs through the minor papilla. Due to the minor papilla’s small size, this results in increased pressure within the pancreatic duct, which can eventually cause pancreatitis [11]. Bertin et al. found that the prevalence of pancreas divisum is similar in patients with idiopathic pancreatitis and in control subjects, suggesting that pancreas divisum by itself is not a direct cause of pancreatitis. They proposed that pancreas divisum might serve as a cofactor in individuals with genetic pancreatitis, particularly those with CFTR mutations [12]. Our patient’s CFTR workup was negative for any abnormalities. This case highlights the importance of maintaining a high index of suspicion for anatomical variants in patients with idiopathic or recurrent pancreatitis, especially when standard evaluations fail to reveal an etiology. It also emphasizes the limitations of relying solely on ERCP for diagnosis, particularly when the anatomy is distorted by ongoing inflammation or necrosis. Furthermore, it demonstrates that even with negative genetic testing and extensive workup, anatomical variants like pancreas divisum can be the root cause of disease and should be considered early in the diagnostic algorithm.

ERCP is the gold standard for diagnosing pancreas divisum, though it is invasive, requires sedation, and carries a complication rate of 10% to 15%, including up to 10% risk of post-ERCP pancreatitis. Diagnosis via ERCP involves cannulating the minor papilla to visualize the dorsal pancreatic duct in 90% to 95% of cases, especially with the use of needle-tip endoscopic catheters [13]. Alternatively, MRCP offers a non-invasive diagnostic option, with secretin-enhanced MRCP (S-MRCP) providing improved visualization. MRCP has a sensitivity of 52% and specificity of 97%, while S-MRCP has a sensitivity of 85% and specificity of 97%. Given these advantages, MRCP and S-MRCP are recommended as the initial diagnostic methods for pancreas divisum, with ERCP reserved for cases where diagnosis is uncertain or for treating symptomatic patients [14]. In our patient, the diagnosis was made via MRCP. He underwent three ERCPs before the MRCP, none of which were diagnostic for pancreas divisum. Due to the patient’s recurrent pancreatitis episodes, his pancreatic anatomy was severely distorted, with findings of necrotizing pancreatitis. This contributed to the delay in diagnosis and obscured the underlying cause.

Currently, ERCP, which may include papillotomy of the minor papilla with or without plastic stent placement, is the preferred therapeutic approach for symptomatic pancreas divisum due to its high efficacy and relatively low complication rate [15]. Systematic reviews show that response rates to ERCP vary by condition, with overall rates of 81.2%, 68.8%, and 53.1% for recurrent acute pancreatitis, chronic pancreatitis, and chronic abdominal pain, respectively, as reported in studies between 1950 and 2008. A larger 2016 study by Lu et al. found an overall response rate of 62.32% with procedures such as endoscopic pancreatic sphincterotomy and ductal stone extraction. Reported complication rates included post-endoscopy pancreatitis (9.93%), infection (3.55%), and hemorrhage (0.71%) [16]. Michailidis et al. confirmed that endoscopic efficacy in pancreas divisum is 67.5% [17]. Additionally, if plastic stent placement fails, removable fully covered self-expandable metal stents have shown a 90% success rate at three months [18]. In our patient, the AXIOS stent failed to provide relief from chronic abdominal pain, and he underwent stent replacement with double pigtail stents.

Surgery is considered for symptomatic pancreas divisum when endoscopic treatments fail, particularly in patients with a normal pancreas or chronic pancreatitis with local complications such as common bile duct stricture or main pancreatic duct stenosis. The surgical approach focuses on addressing these complications and improving pancreatic drainage. Common procedures include sphincteroplasty, duodenopancreatectomy, and duodenum-preserving pancreatic head resection. Recent meta-analyses suggest that surgical intervention may offer superior outcomes compared to endoscopic treatments, with higher success rates and lower complication and re-intervention rates [19]. Despite these findings, the evidence remains mixed due to variability in study quality. Therefore, surgery should be considered selectively and primarily when endoscopic approaches fail. Further research is needed to refine selection criteria for surgical interventions. Our patient’s symptoms have improved, and he is currently doing well.

## Conclusions

This case highlights the challenges of diagnosing pancreas divisum in patients with recurrent pancreatitis. Despite multiple admissions and interventions, the anatomical defect was only identified during the eighth hospitalization, underscoring the limitations of conventional diagnostic approaches. Earlier use of advanced imaging and a higher index of suspicion for anatomical anomalies could have expedited diagnosis and prevented recurrent hospitalizations. By presenting this case, we aim to raise awareness of the clinical consequences of delayed diagnosis in pancreas divisum and to advocate for earlier consideration of non-invasive imaging modalities such as S-MRCP in similar clinical scenarios. We believe this case provides valuable insights for physicians managing complex cases of recurrent pancreatitis and supports the need for a multidisciplinary and stepwise diagnostic approach.
